# Respiratory syncytial virus (RSV) attachment and nonstructural proteins modify the type I interferon response associated with suppressor of cytokine signaling (SOCS) proteins and IFN-stimulated gene-15 (ISG15)

**DOI:** 10.1186/1743-422X-5-116

**Published:** 2008-10-13

**Authors:** Elizabeth C Moore, Jamie Barber, Ralph A Tripp

**Affiliations:** 1 Department of Infectious Diseases, Center for Disease Intervention, University of Georgia, Athens, GA 30602, USA

## Abstract

Respiratory syncytial virus (RSV) is a major cause of severe lower airway disease in infants and young children, but no safe and effective RSV vaccine is yet available. Factors attributing to this problem are associated with an incomplete understanding of the mechanisms by which RSV modulates the host cell response to infection. In the present study, we investigate suppressor of cytokine signaling (SOCS)-1 and SOCS3 expression associated with the type I IFN and IFN-stimulated gene (ISG)-15 response following infection of mouse lung epithelial (MLE-15) cells with RSV or RSV mutant viruses lacking the G gene, or NS1 and NS2 gene deletions. Studies in MLE-15 cells are important as this cell line represents the distal bronchiolar and alveolar epithelium of mice, the most common animal model used to evaluate the host cell response to RSV infection, and exhibit morphologic characteristics of alveolar type II cells, a primary cell type targeted during RSV infection. These results show an important role for SOCS1 regulation of the antiviral host response to RSV infection, and demonstrate a novel role for RSV G protein manipulation of SOCS3 and modulation of ISG15 and IFNβ mRNA expression.

## Background

Respiratory syncytial virus (RSV), a member of the *Pneumovirus *genus within the family *Paramyxoviridae*, is the single most important viral respiratory pathogen infecting infants and young children worldwide, as well as an important cause of respiratory tract illness in the elderly, transplant patients, and immune suppressed [12,22,33,48,51]. The RSV genome (15 kb) is single-stranded, negative-sense RNA that contains 10 transcription units which are sequentially transcribed to produce 11 proteins in the following order: NS1, NS2, N, P, M, SH, G, F, M2-1, M2-2, and L [52]. The NS1 and NS2 non-structural proteins are not expressed on the virion but are two of the most abundantly expressed RNAs in RSV-infected cells due to their promoter-proximal location [5,11,15] These accessory proteins have been shown to act cooperatively to suppress the activation and nuclear translocation of the IFN-regulatory factor IRF-3 [4,47], and inhibit the type I IFN signaling cascade by mediating proteosome degradation of signal transducer and activator of transcription 2 (STAT2) with Elongin-Cullin E3 ligase [10,29].

Additionally, constructs of "humanized" NS1 and NS2 recombinant protein expressed in *Escherichia coli *have been shown to decrease STAT2 levels as well as type I IFN responsiveness [29], and recent RNA interference (RNAi) studies in mice targeting NS proteins for silencing by short interfering RNA (siRNA) resulted in inhibition of RSV replication in mice [67]. The NS1 and NS2 proteins may also function to facilitate RSV replication outside the interferon arena as they have an anti-apoptotic effect on RSV-infected A549 cells thereby enhancing viral replication [3].

Increasing evidence suggests that other RSV proteins, particularly the surface proteins on the virion, have important roles in facilitating RSV infection and replication [51]. The RSV surface attachment protein, i.e. G protein, has been shown to modify pulmonary trafficking of immune cells [55], as well as the pattern and type of cytokine and chemokine expression by bronchoalveolar leukocytes (BAL) and bronchoepithelial cells in RSV-infected mice [53,55] and in RSV-infected humans [2,23,49]. The G protein has been shown to have a CX3C chemokine motif in the central conserved region of the protein that can mimic some of the activities of fractalkine, the only known CX3C chemokine, specifically binding to CX3CR1 and mediating CX3C-CX3CR1 leukocyte chemotaxis [16,54]. Importantly, anti-G protein antibody responses after recent RSV infection or vaccination in humans are associated with inhibition of RSV G protein CX3C-CX3CR1 interaction and G protein-mediated leukocyte chemotaxis [17].

The G protein has also been shown to inhibit Toll-like receptor (TLR) 3/4-mediated IFN-beta induction [45], a feature that may facilitate virus replication. Interestingly, the RSV F protein has been shown to induce aspects of innate immunity through TLR4 signaling [28], and TLR4-deficient mice challenged with RSV exhibit impaired NK cell and CD14^+ ^cell pulmonary trafficking, deficient NK cell function, impaired interleukin-12 expression, and impaired virus clearance compared to mice expressing TLR4 [18]. In addition, TLR4 polymorphisms in humans are linked to impaired responses to respiratory syncytial virus [59] and the genetic predisposition to severe RSV infection [39]. These features appear contradictory to facilitating RSV replication, but F protein activation of TLR signaling may be an important feature to desensitize TLR activation of immunity. For example, RSV has been shown to mediate long-term desensitization of lung alveolar macrophages to TLR ligands [8]. This feature may be linked to the lack of durable protective immunity associated with RSV infection [50,51]. Finally, the RSV SH protein is linked to altered Th1-type cytokine and chemokine expression by BAL cells [55], and can inhibit TNFα signaling [13]. Taken together, RSV surface proteins have immune modulatory features that appear to facilitate infection and replication.

It is not surprising that TLRs have an important role in the host response to RSV infection. Viral infection has been shown to activate TLRs and retinoic acid inducible gene I (RIG-I) signaling pathways leading to phosphorylation of interferon regulatory factor3 (IRF3) and IRF7 and stimulation of type I interferon (IFN) transcription, a process important for innate antiviral immunity [26]. Production of type I IFN depends on activation of IRF3 and IRF7 [20,35,44] where type I IFN expression is negatively regulated by suppressor of cytokine signaling (SOCS) proteins [7,24]. SOCS proteins are mainly regulated at the transcriptional level but can be directly induced by stimulation of TLRs where they do not interfere with direct TLR signaling, but instead regulate paracrine IFN signaling [7]. The SOCS protein family is comprised of eight proteins (CIS, cytokine-inducible SH2-containing protein, SOCS1-7) of structural and functional homology [7,24]. Of the family members, SOCS1 and SOCS3 appear to be the most effective in regulating type I IFN expression. SOCS1 can directly associate with high affinity to all Janus kinases (JAKs) directly inhibiting their catalytic activity, while SOCS3 functions in part by interacting with activated cytokine receptors [10].

Numerous studies have established that type I IFN expression regulates hundreds of host genes that include STAT1, JAK1, ERK1, MxA, RIG-I, and IRF3 [9,14,27,30,32,68]. One important IFN-stimulated gene that encodes an ubiquitin-like protein is IFN-stimulated gene (ISG)-15 (ISG15). ISG15 is one of the earliest ISG induced by type I IFN and has been shown to target several components of the antiviral signaling pathway [27].

Virally-induced ISG15 promotes an antiviral state by subverting proteosome-mediated degradation of IRF3 in infected cells [38]. As for type I IFNs, viruses have adapted to circumvent the antiviral effects of ISG15. One example is the ability of the NS1 protein of the influenza B virus to inhibit conjugation of ISG15 to target proteins [65]. Since IFN genes are generally transcriptionally silent until induced, for example by binding of TLR-activated transcription factors to their promoters, ISG15 expression can reveal pathogen-TLR activation of the type I IFN response.

RSV infects ciliated airway epithelial cells in the respiratory tract [19,66] and type II pneumocytes [6,36,58,60,61]. A majority of RSV studies have used the mouse model to evaluate the host response to infection. This model has been useful to understand aspects of the immunobiology of infection. Mouse lung epithelial (MLE)-15 cells offer a good option to emulate the mouse model of RSV infection as these cells are a type II pneumocyte cell line representing the distal bronchiolar and alveolar epithelium that maintain their differentiated phenotypes and functional characteristics for up to 30–40 cell culture passages [63]. MLE-15 cells also express microvilli, SP-A, SP-B and SP-C, form basement membranes, and are capable of expressing MHC class I antigens [34,63,69]. In general, type II pneumocytes comprise approximately 15% of total lung cells, and are found at the air-liquid interface [37,64]. From this position, type II pneumocyte cells are able to respond to airborne stimuli as well as interact with various immune cells such as CD8^+ ^T cells which are known to be important immune mediators of respiratory viral infections.

The studies reported here focus on the early antiviral host response in MLE-15 cells to RSV infection and the role of RSV surface proteins in modulating this response. The studies center on SOCS1 and SOCS3 negative regulation of the type I IFN response and ISG15 expression following infection with RSV or RSV mutant viruses lacking the G gene, or NS1 and NS2 gene deletions. These results indicate an important role for SOCS1 regulation of the antiviral host response to RSV infection, and reveal a novel role for RSV G protein modulation of SOCS3, ISG15 and IFNβ mRNA expression.

## Results

### RSV stimulation of SOCS1, SOCS3, IFNα and IFNβ mRNA expression

To determine the relationship between RSV infection, RSV proteins, and SOCS regulation of the type I IFN response, MLE-15 cells were infected with RSV (WT) or RSV mutant viruses lacking both the NS1 and NS2 genes(ΔNS1/2) or the G gene (ΔG). The level of RSV and RSV mutant virus replication in MLE-15 cells infected at a multiplicity of infection (MOI) = 1.0 at 24 and 48 h post-infection (pi) was determined byquantitative real-time PCR analysis of RSV nucleocapsid (N) gene expression. At 24 h pi, the level of virus replication was similar between RSV and RSV mutant viruses where N gene copies were 2.6 × 10^5 ^for WT, 2.1 × 10^5 ^for ΔNS1/2, and 2.7 × 10^5 ^for ΔG viruses. However, at 48 h pi, the level of ΔNS1/2 virus replication was significantly (p < 0.01) lower (6.4 × 10^4 ^N gene copies) compared to RSV (5.5 × 10^5 ^N gene copies) or ΔG (4.9 × 10^5 ^N gene copies) virus replication which was not significantly (p < 0.05) different from each other. Visual examination of RSV and RSV mutant virus infected MLE-15 cells at 48 h pi showed higher cytopathic effects for ΔNS1/2 infected cells compared to RSV or ΔG infected MLE-15 cells. These findings are consistent with the report showing RSV nonstructural proteins have an important role in delaying apoptosis linked to infection [3].

**Figure 1:**
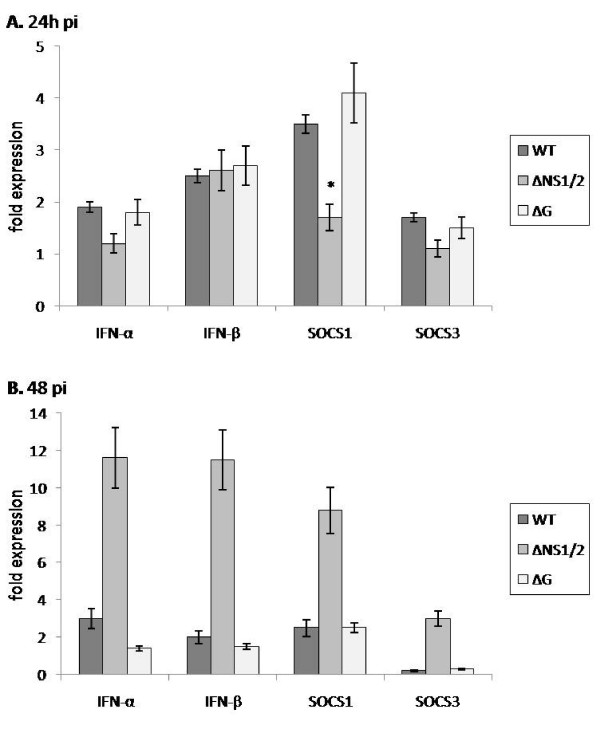
**RSV stimulation of SOCS1, SOCS3, IFNα and IFNβ mRNA expression.** MLE-15 cells were mock-infected or infected with WT, ΔG, or ΔNS1/2 virus at a multiplicity of infection (MOI) of 1 for 24 h (A) or 48 h (B). Cells were harvested at the times indicated. SOCS1, SOCS3, IFNα and IFNβ mRNA expression were measured by real-time PCR. Transcript levels were normalized to hypoxanthine guanine phosphoribosyl transferase (HPRT) expression and calibrated to the mock condition. Data is presented as fold-differences in gene expression relative to mock-infected MLE-15 cells. Differences in gene fold expression between virus infection groups were evaluated by Mann-Whitney U test and noted as significant as denoted by an asterisk. Data are shown as means ± standard errors (SE) of the means.

RSV and RSV mutant virus infection of MLE-15 cells at 24 h pi was associated with IFNα, IFNβ and SOCS1 and SOCS3 mRNA expression. SOCS1 mRNA expression was significantly (p < 0.01) lower in ΔNS1/2 virus infected MLE-15 cells compared to WT or ΔG virus infected cells (Figure 1A). This finding is in keeping with the findings of NS1/NS2 antagonism of type I IFNs [4,46,47] and suggests the possibility that type I IFN antagonism is linked to NS1/NS2 induction of SOCS1 and subsequent negative regulation of type I IFN activity [7,24]. The level of SOCS3 mRNA expression was similar in WT, ΔG or ΔNS1/2 virus infected MLE-15 cells. Since the level of virus replication was similar between RSV and RSV mutant viruses at 24 h pi, and SOCS1 mRNA expression was significantly lower in ΔNS1/2 virus infected MLE-15 cells, these results suggest that RSV infection of MLE-15 cells preferentially induces SOCS1 over SOCS3 mRNA expression, an effect associated with NS1/NS2 expression.

Despite differences in SOCS1 mRNA expression, the levels of IFNα and IFNβ mRNA expression were similar between RSV and RSV mutant virus infected MLE-15 cells. This is not unexpected because SOCS proteins form part of a classical negative feedback loop that is time-dependent [24], thus RSV and RSV mutant virus infection of MLE-15 cells and IFNα, IFNβ and SOCS1 and SOCS3 mRNA expression was examined at 48 h pi.

At 48 h pi, ΔNS1/2 virus infected MLE-15 cells had significantly (p < 0.01) higher IFNα and IFNβ mRNA expression compared to WT or ΔG virus infected cells (Figure 1B), indicating a governing function of NS1/NS2 in type I IFN antagonism. In addition, a higher level of SOCS1 mRNA expression was evident at 48 h pi compared to similar infection at 24 h pi (Figure 1A) despite a significantly (p < 0.05) lower N gene copy compared to WT or ΔG virus infected cells.

The higher SOCS1 mRNA expression at 48 h pi possibly reflects a compensating host cell mechanism to regulate type I IFN expression as SOCS3 mRNA expression also increased. The levels of IFNα and IFNβ and SOCS1 and SOCS3 mRNA expression were similar between WT and ΔG virus infected MLE-15 cells. Comparing time-points post-WT or ΔG virus infection, no significant (p < 0.05) changes in IFNα, IFNβ or SOCS1 mRNA expression were observed at 24 h pi (Figure 1A) or 48 h pi (Figure 1B); however, SOCS3 mRNA expression was considerably decreased from 24 h pi to 48 h pi.

### RSV stimulation of SOCS1, SOCS3, IFNα and IFNβ protein expression

**Figure 2:**
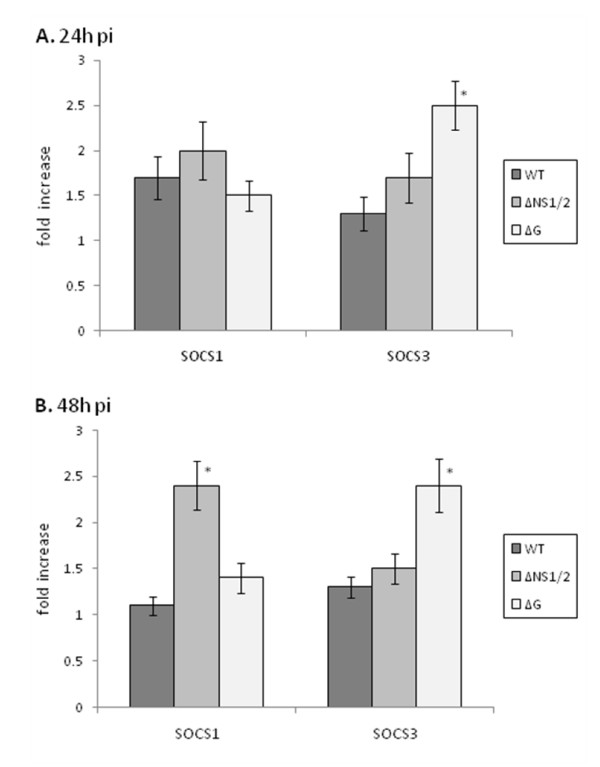
**RSV stimulation of SOCS1 and SOCS3 protein expression.** RSV stimulation of SOCS1 and SOCS3 protein expression was determined in MLE-15 cells that were mock-infected or infected with WT, ΔG, or ΔNS1/2 virus at a multiplicity of infection (MOI) of 1 for 24 h (A) or 48 h (B). Cells were harvested at the times indicated and intracellular SOCS1 or SOCS3 levels determined by flow cytometry. Data is presented as fold-differences in protein expression relative to mock-infected cells. Differences in fold expression between virus infection groups were evaluated by Mann-Whitney U test and noted as significant as denoted by an asterisk. Data are shown as means ± standard errors (SE) of the means.

To determine if the type I IFN and SOCS mRNA expression profiles in RSV and RSV mutant virus infected cells were reiterated by protein expression, intracellular IFNα, IFNβ and SOCS1 and SOCS3 protein levels were determined at 24 h and 48 h pi by flow cytometry (Figure 2). At 24 h or 48 h pi, IFNα and IFNβ protein expression in RSV and RSV mutant virus infected MLE-15 cells was low and not readily detected. In the mouse, total IFNα is comprised of at least 14 IFNα genes and 3 IFNα pseudogenes [57], and because the quantity of IFNα measured depends on the specificity of the detection antibody for these isoforms, detection of IFNα is limited. Moreover, low levels of type I interferon protein expression would be predicted in part because of the transient nature of these proteins as they are rapidly secreted and their expression is regulated by factors linked to IFN-stimulated genes such as ISG15 which targets several components of the IFN signaling pathway [27,62]. At 24 h pi, SOCS1 protein expression levels were similar following infection with RSV or RSV mutant viruses; however SOCS3 protein expression was significantly (p < 0.05) higher in ΔG virus infected cells compared to WT infected cells, and substantially higher compared to ΔNS/2 virus infected cells (Figure 2A). The higher SOCS3 protein expression following ΔG virus infection suggests that G protein expression reduces SOCS3 protein expression during RSV infection. This may be important to enhance SOCS-mediated negative regulation of cytokine expression [7,24] and/or alter the Th1/Th2 cell differentiation process to facilitate virus replication, as SOCS3 has been linked to the development of Th2-type responses [25]. At 48 h pi, ΔNS1/2 virus infected cells expressed significantly higher (p < 0.05) SOCS1 protein compared to WT and ΔG virus infected MLE-15 cells (Figure 2B), a finding consistent with SOCS1 mRNA expression at 48 h pi (Figure 1B), and the concept that NS1/NS2 proteins mediate IFN antagonism in part by affecting SOCS1 negative regulation of type I IFN activity [7,24].

Similar to the 24 h pi finding, at 48 h pi ΔG virus infected cells expressed significantly (p < 0.05) higher SOCS3 protein compared to WT or ΔNS1/2 infected cells (Figure 2B). Since NS1/NS2 in RSV has been shown to act cooperatively to suppress the activation and nuclear translocation of the IFN-regulatory factor IRF-3 [4,47], and antagonize type I IFN activity by inhibiting the type I IFN and the signaling cascade [10,29], the results indicate that SOCS3 may not have an essential role governing type I IFN during RSV infection, but may have an ancillary role to facilitate virus replication.

### RSVΔG virus infection mediates enhanced IFNβ secretion

Intracellular type I IFN expression in RSV and RSV mutant virus infected MLE-15 cells was not effectively detected above background levels at 24 h and 48 h pi by flow cytometry.

**Figure 3:**
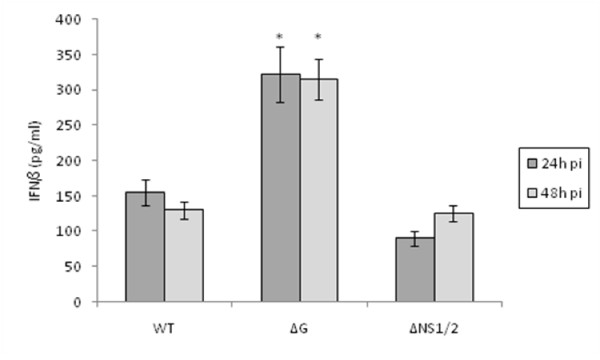
**RSVΔG virus infection mediates enhanced IFNβ secretion.** The levels of IFNβ in MLE-15 cell culture supernatant were determined following infection with WT, ΔG, or ΔNS1/2 virus at a multiplicity of infection (MOI) of 1 for 24 h (A) or 48 h (B) as indicated. Data are shown as means ± standard errors (SE) of the means.

Commercially available mouse IFNα ELISA kits were evaluated but found to have a poor threshold of detection as expected given the limited specificity of the detection antibody used in the kits for detection of the numerous IFNα isoforms [57]. However, IFNβ was detected in all RSV and RSV mutant virus infected MLE-15 cell culture supernatants (Figure 3).

MLE-15 cells infected with ΔG virus had significantly (p < 0.01) higher levels of IFNβ compared to WT or ΔNS1/2 virus infected cells at 24 h and 48 h pi, indicating that G protein expression inhibits IFNβ protein expression. RSV has been shown to down-regulate STAT2 protein expression [10] and the type I IFN JAK-STAT pathway [40], thus it is possible that G protein inhibits cellular transcription factors involved in IFNβ signaling. IFNβ levels in the supernatant from ΔNS1/2 virus infected cells was slightly but insignificantly lower compared to cell culture supernatant from WT virus infected cells.

### ISG15 expression is increased in the absence of G protein expression

**Figure 4:**
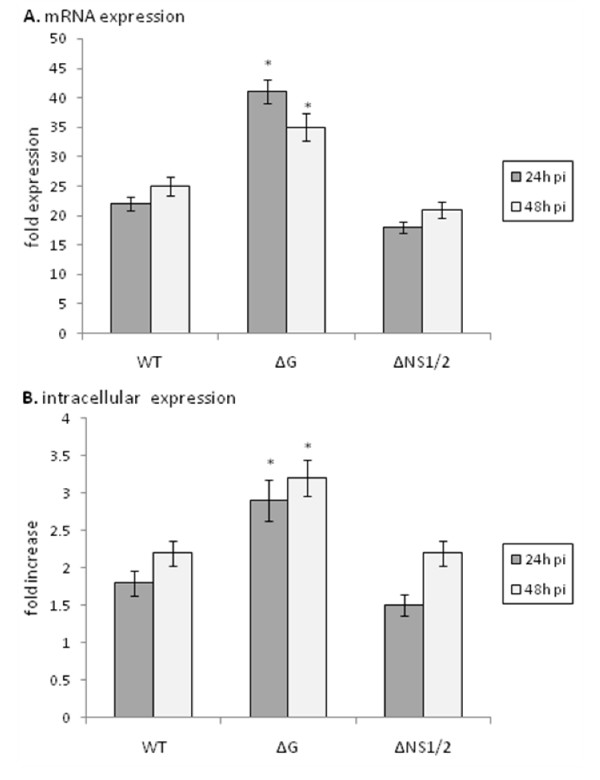
**ISG15 expression is increased in the absence of G protein expression.** MLE-15 cells were mock-infected or infected with WT, ΔG, or ΔNS1/2 virus at a multiplicity of infection (MOI) of 1 for 24 h or 48 h as indicated. ISG15 message expression was measured by real-time PCR (A). Transcript levels were normalized to hypoxanthine guanine phosphoribosyl transferase (HPRT) expression and calibrated to the mock condition. (B) RSV stimulation of ISG15 protein expression was determined in MLE-15 cells that were mock-infected or infected with WT, ΔG, or ΔNS1/2 virus at a multiplicity of infection (MOI) of 1 for 24 h or 48 h as indicated. Cells were harvested and ISG15 levels determined by flow cytometry. Data is presented as fold-differences in protein expression relative to mock-infected cells. Differences in fold expression between virus infection groups were evaluated by Mann-Whitney U test and noted as significant as denoted by an asterisk. Data are shown as means ± standard errors (SE) of the means.

Expression of the interferon-stimulated gene, ISG15, was determined in RSV and RSV mutant virus infected MLE-15 cells (Figure 4). ISG15 has been shown to modify several important molecules linked to and affecting type I interferon signal transduction, is released from cells to mediate extracellular cytokine-like activities, and evidence suggests that IFNβ and ISG15 are induced in parallel as a primary response to infection [1,38,41,42]. The level of ISG15 mRNA expression (Figure 4A) was similar to the level of ISG15 protein expression at 24 h and 48 h pi where similar levels were observed following WT or ΔNS1/2 infection of MLE-15 cells.

However, ISG15 mRNA (Figure 4A) and protein (Figure 4B) levels were significantly (p < 0.05) higher in ΔG virus infected cells compared to WT or ΔNS1/2 virus infected cells indicating that G protein expression impedes ISG15 mRNA and protein expression. These findings are consistent with IFNβ governance of ISG15 expression [1,38,41,42], and the finding that G protein expression inhibits IFNβ protein expression (Figure 3).

## Discussion

Numerous studies investigating the host cell response associated with RSV infection have shown that RSV proteins can affect the spectrum of the antiviral cytokine response [2,15,31,36,51,56], but the mechanisms linked to RSV protein regulation of the associated cell signaling pathway remains unclear. The studies reported here examine the early antiviral host response in MLE-15 cells to RSV infection and the role of RSV surface proteins in modulating this response. Studies in MLE-15 cells are important as this cell line represents the distal bronchiolar and alveolar epithelium of mice [63], and mice are the most common animal model used to evaluate the host cell response to RSV infection. MLE-15 cells exhibit morphologic characteristics of alveolar type II cells that include microvilli, cytoplasmic multi-vesicular bodies, and multi-lamellar inclusion bodies, maintain functional characteristics of distal respiratory epithelial cells including the expression of surfactant proteins [63], thus using MLE-15 cells as a proxy for RSV infection in mice offers several advantages to advance studies examining the host cell response to infection. In these studies, the role of SOCS1 and SOCS3 negative regulation of the type I IFN response and ISG15 expression were evaluated after infection of MLE-15 cells with RSV or RSV mutant viruses lacking the G gene, or having NS1 and NS2 gene deletions. RSV and RSV mutant virus infection of MLE-15 cells induced different type I IFN and SOCS1 and SOCS3 mRNA expression patterns at 24 h and 48 h pi, a feature that may be linked to sequential RSV gene expression due to their promoter-proximal location in the genome [5,11,15]. At 24 h pi, SOCS1 mRNA expression was significantly lower in ΔNS1/2 virus infected MLE-15 cells compared to WT or ΔG virus infected cells. This finding is consistent with NS1/NS2 antagonism of type I IFN activity [4,46,47]. These results also indicate that NS1/NS2 may in part mediate type I IFN antagonism through the induction of SOCS1 which negatively regulates type I IFN expression [7,24]. At 48 h pi, SOCS1 mRNA and protein expression was higher in ΔNS1/2 virus infected MLE-15 cells compared to WT or ΔG virus infected cells suggesting a host cell compensating mechanism to negatively regulate an earlier increase in type I IFN expression or cell signaling.

Interestingly, SOCS3 protein expression was significantly higher in MLE-15 cells infected with ΔG virus compared to WT or ΔNS1/2 virus infected cells, indicating that G protein expression deters SOCS3 protein expression during RSV infection. Since SOCS3 is predominantly expressed during the Th2-type immune response and reciprocally inhibits Th1-type differentiation processes [25], the results suggest that G protein may induce SOCS3 protein expression to facilitate RSV replication by inhibiting antiviral Th1-type responses.

Several factors negatively regulate IFNβ, and for RSV, it has been recently shown that RSV G proteins mediates down-regulation of IFNβ by inhibiting IFNβ promoter activation [45], demonstrating yet another novel function of the G protein in the regulation of host cell response. In the study reported here, significantly higher levels of IFNβ expression were detected in the cell culture supernatants of ΔG virus infected MLE-15 cells compared to WT or ΔNS1/2 virus infected cells, a finding consistent with the G protein inhibition of IFNβ promoter activation [45]. No increase in IFNβ expression was detected in the cell culture supernatant of ΔNS1/2 virus infected MLE-15 cells relative to WT virus infected cells despite the reported finding that NS1 and NS2 act cooperatively to suppress activation and nuclear translocation of IRF3 [47]. Since RSV-induced cytokine gene expression occurs through the activation of a subset of transcription factors including IRF3 [21], the ability of RSV to induce expression and catalytic activity IKKε which blocks RSV-induced IRF3 phosphorylation, nuclear translocation and DNA-binding, and leading to inhibition of cytokine gene transcription, mRNA expression and protein synthesis [21] may mask the activities of NS1/NS2.

Interferon stimulated gene (ISG)-15 is a type I interferon-induced molecule that is rapidly upregulated in response to viral infection [38,41]. Expression of ISG15 mRNA and protein expression was significantly upregulated in the absence of the RSV G gene (ΔG virus) at 24 h and 48 h pi indicating the novel finding that G protein modifies ISG15 expression to limit its role in the antiviral host cell response. ISG15 is one of scores of ISGs which may be induced directly or indirectly by virus proteins or byproducts of virus infection [43]; however, as expression of ISG15 mRNA and protein was similar between ΔNS1/2 and WT virus infection of MLE-15 cells, it is unlikely NS1/NS2 has a role in modifying ISG15. The finding in this study that G protein expression inhibits IFNβ and ISG15 protein expression is consistent with evidence suggesting that IFNβ and ISG15 are induced in parallel as a primary response to infection [1,38,41,42], and that this pathway is targeted by RSV G protein.

## Conclusion

The findings from this study show an important role for SOCS1 regulation of the early type I IFN response to RSV infection, and allude to the possibility that NS1/NS2 may in part mediate type I IFN antagonism through the induction of SOCS1 negative regulation of type I IFN expression. In addition, the results show that RSV G protein has reduced SOCS3 expression and shows a previously unrecognized role of G protein in regulation of IFNβ and ISG15 expression.

Notably, these studies were performed using MLE-15 cells, a type II alveolar cell line that represents the distal bronchiolar and alveolar epithelium of mice, the most common animal model used to evaluate the host cell response to RSV infection. Thus, these findings have important implications in understanding the mechanisms linked to RSV disease pathogenesis and treatment.

## Methods

### Viruses and cells

Type I IFN-free virus stocks of recombinant RSV strain A2 (6340WT), 6340WT lacking the G protein gene (6340 G), and 6340WT lacking NS1 and NS2 genes (ΔNS1/2) (kind gift of Peter Collins, NIH) were propagated in Vero cells (African green monkey kidney fibroblasts, ATCC CCL 81) maintained in DMEM (Sigma-Aldrich Corp., St. Louis, MO, USA) supplemented with 5% heat-inactivated (56°C) fetal bovine serum (FBS; Hyclone Laboratories, Salt Lake City, Utah) as previously described [55]. Infectious virus titers were determined on Vero cells by endpoint dilution and counting of infected cell foci stained for indirect immunofluorescence with an RSV F-specific monoclonal antibody (clone 131-2A) as previously described [55].

Mouse lung epithelial (MLE)-15 cells (kind gift from Dr. Jeffrey A. Whitsett, Children's Hospital Medical Center, Cincinnati, Ohio) are an immortalized type II pneumocyte cell line representing the distal bronchiolar and alveolar epithelium that maintain their differentiated phenotypes and functional characteristics for up to 30–40 cell culture passages. MLE-15 cells were propagated in hydrocortisone-insulin-transferrin-β-estradiol-sodium selenite (HITES) medium supplemented with 4% fetal bovine serum as previously described [63].

### RNA isolation and quantitative real-time PCR

Total RNA was isolated from uninfected, uninfected Vero cell lysate treated, and RSV and RSV mutant virus infected (MOI = 1) MLE-15 cells at 24 h or 48 h pi using RNeasy Mini kit (Qiagen, Valencia, CA) and stored at -80°C until used. Reverse transcription of pooled RNA was performed using random hexamers and MuLV reverse transcriptase (Applied Biosystems, Foster City, CA). cDNA diluted 1:4 was used as template using SOCS1, SOCS3, pooled IFNα4 and IFNα9, and IFNβ1 gene expression assays (Applied Biosystems, Foster City, CA) and analyzed using MX300P software by Stratagene (La Jolla, CA). Each gene of interest was normalized to hypoxanthine guanine phosphoribosyl transferase (HPRT) expression and calibrated to its corresponding expression in mock-infected or mock-stimulated MLE-15 cells. Data is presented as fold-differences in gene expression relative to mock-infected or mock-stimulated MLE-15 cells.

To establish a standard curve for the quantitation of RSV N gene present in RSV-infected MLE-15 cells, the RSV N gene was amplified by PCR and inserted into a pcDNA3.1 vector. This vector was then used to transform competent E. coli One Shot^® ^TOP10 cells (Invitrogen, Carlsbad, CA). The colonies were screened for ampicillin resistance and the resulting plasmid containing the RSV N gene was verified by sequence analysis. The standard curve was created using 10-fold serial dilutions of 1 ug/ul of RSV N gene plasmid. Samples along with standard curve dilutions were analyzed by real-time PCR with the Stratagene Mx3000P or Mx3005P for 40 cycles with custom RSV N gene primers purchased from Applied BioSystems. Data is expressed as copies of RSV N gene.

### Intracellular protein analysis by flow cytometry

MLE-15 cells were infected with WT, ΔG or ΔNS1/2 virus at a MOI = 1.0, mock infected with uninfected Vero cell lysate, or incubated in the presence of media alone. At 24 and 48 hours pi, the cells were treated with 1μg/ml BD GolgiPlug™ (Brefeldin A, BD Pharmingen, San Diego, CA) for 5 hours prior to fixation with 4% formaldehyde and analyzed or stored at 4°C prior to staining. Cells were permeabilized with 1× BD Perm/Wash™ and stained with either rabbit anti-SOCS1 polyclonal antibody or goat anti-SOCS3 polyclonal antibodies (Abcam, Cambridge UK), rabbit anti-ISG15 polyclonal antibody (Cell Signaling Technology, Danvers, MA) or rat anti-mouse IFNα or IFNβ polyclonal antibody (PBL InterferonSource, Piscataway, NJ) using similar methods as previously described [55]. Intracellular protein expression was analyzed using a BD LSR II flow cytometer and evaluating 30,000 gated events. Data is presented as fold increase relative to cells cultured in the presence of media only.

### ELISA quantitation in cell supernatants

MLE-15 cells were infected with WT, ΔG or ΔNS1/2 virus at a MOI = 1.0, mock infected with uninfected Vero cell lysate, or incubated in the presence of media alone. At 24 and 48 hours pi, cells supernatants were collected, centrifuged to remove potential cell contamination and debris, and used immediately or stored at -80°C prior to analysis. Levels of IFNβ in cell culture supernatants were measured using the Mouse Interferon Beta ELISA kit (PBL Biomedical Laboratories, Piscataway, NJ) according to the manufacturer's protocol. Absorbance at 450 nm was read using the BIO-TEK PowerWave XS microplate reader (Tecan US, Durham, NC) and the data was analyzed using KC junior software (Tecan US, Durham, NC).

### Statistics

All experiments in this study were independently performed 5–6 times. For PCR assays, differences in gene fold expression were evaluated by Student *t *test and considered significant when the *P *value was <0.05. Data are shown as means ± standard errors (SE) of the means. Comparison of results between RSV and RSV mutant virus experiments were performed by the Mann-Whitney U test using the InStat 3.05 biostatistics package (GraphPad, San Diego, CA). Unless otherwise indicated, mean ± SEM is shown.

## Competing interests

The authors declare that they have no competing interests.

## Authors' contributions

EM carried out the molecular studies, cell studies and ELISA assays, participated in the flow cytometry, and drafted the manuscript. JB performed the flow cytometry. RT conceived the study, participated in the design of the study, and with EM performed the statistical analysis. All authors read and approved the final manuscript.
